# Correction: Javed et al. The Potential Impact of Smog Spell on Humans’ Health Amid COVID-19 Rages. *Int. J. Environ. Res. Public Health* 2021, *18*, 11408

**DOI:** 10.3390/ijerph192316269

**Published:** 2022-12-05

**Authors:** Ammar Javed, Farheen Aamir, Umar Farooq Gohar, Hamid Mukhtar, Muhammad Zia-UI-Haq, Modhi O. Alotaibi, May Nasser Bin-Jumah, Romina Alina Marc (Vlaic), Oana Lelia Pop

**Affiliations:** 1Institute of Industrial Biotechnology, Government College University Lahore, Lahore 54000, Pakistan; 2Office of Research, Innovation & Commercialization, Lahore College for Women University, Lahore 54000, Pakistan; 3Biology Department, College of Science, Princess Nourah Bint Abdulrahman University, Riyadh 11671, Saudi Arabia; 4Environment and Biomaterial Unit, Health Sciences Research Center, Princess Nourah Bint Abdulrahman University, Riyadh 11671, Saudi Arabia; 5Food Engineering Department, Faculty of Food Science and Technology, University of Agricultural Sciences and Veterinary Medicine, 400372 Cluj-Napoca, Romania; 6Department of Food Science, University of Agricultural Science and Veterinary Medicine, 400372 Cluj-Napoca, Romania

There was an error in the original publication [1]. **There were typo errors on page 9 and 12, along with self-citations in references 6, 17, 21, 32, 42, 49, 56, 57, 62 and 74**.

## Text Correction

A correction has been made to ** 5. COVID-19 Pandemic**:

Page 9 first paragraph: The 2019 novel COVID for what it’s worth presently called, has quickly spread globally [70].

Page 9 second paragraph: Wuhan, Hubei region, China and in local workers of the Hunan seafood market should be deleted.

A correction has been made to ** 8. Impact of Lockdown on Smog**:

Page 12 first paragraph: on Wuhan and encompassing urban areas should be deleted.

## References

A correction has been made to References **6, 17, 21, 32, 42, 49, 56, 57, 62 and 74**:

1. Reference 6 should be read as

Roman, M.; Idrees, M.; Ullah, S.; Idrees, M.; Sharif, M.; Street, P.; District, H. A Sociological Study of Environmental Pollution and Its Effects on the Public Health Faisalabad City. Int. J. Educ. Res. 2013, 1, 1–12.

2. Reference 17 should be read as

Tsai, D.H.; Riediker, M.; Berchet, A.; Paccaud, F.; Waeber, G.; Vollenweider, P.; Bochud, M. Effects of short- and long-term exposures to particulate matter on inflammatory marker levels in the general population. Environ. Sci. Pollut. Res. 2019, 26, 19697–19704, doi:10.1007/s11356-019-05194-y.

3. Reference 21 should be read as 

Filippini, T.; Rothman, K.J.; Cocchio, S.; Narne, E.; Mantoan, D.; Saia, M.; Goffi, A.; Ferrari, F.; Maffeis, G.; Orsini, N. Associations between mortality from COVID-19 in two Italian regions and outdoor air pollution as assessed through tropospheric nitrogen dioxide. Sci. Total Environ. 2021, 760, 143355.

4. Reference 32 should be read as 

Reference 32 should be replaced by Jacobs, E.T.; Burgess, J.L.; Abbott, M.B. The Donora Smog Re-visited: 70 Years After the Event That Inspired the Clean Air Act. Am J Public Health. 2018 Apr;108(S2):S85-S88. doi: 10.2105/AJPH.2017.304219.

5. Reference 42 should be read as 

Reference 42 should be replaced by Li, B.; Shi, H.; Yang, D.C.; Peng, M. Smog Pollution, Envi-ronmental Uncertainty, and Operating Investment. Atmosphere 2021, 12, 1378. https://doi.org/10.3390/atmos12111378.

6. Reference 49 should be read as 

Reference 49 should be replaced by Raza, W.; Saeed, S.; Saulat, H.; Gul, H.; Sarfraz, M.; Sonne, C.; Kim, K.-H. (2020). A review on the deteriorating situation of smog and its preventive measures in Pakistan. Journal of Cleaner Production, 123676. doi:10.1016/j.jclepro.2020.123676.

7. Reference 56 should be read as 

Yang, C. Policies, reglatory framework and enforcement for air quality management: The case of China-Environment working paper no.157. Organ. Econ. Co-operation Dev. 2020, 4”

8. Reference 57 should be read as 

Li, Y.; Tang, Y.; Fan, Z.; Zhou, H.; Yang, Z. Assessment and comparison of three different air quality indices in China. Environ. Eng. Res. 2018, 23, 21–27, doi:10.4491/eer.2017.006”.

9. Reference 62 should be read as

Orlando, E. The evolution of EU policy and law in the environmental field: Achievements and current challenges. EU, US Glob. Clim. Gov. 2016, 61–80.

10. Reference 74 should be read as 

Muralidar, S.; Visaga, S.; Sekaran, S. The emergence of COVID-19 as a global pandemic: Understanding the epidemiology, immune response and potential therapeutic targets of SARS-CoV-2. Biochimie 2020, 179, 85–100

The authors apologize for any inconvenience caused, and state that the scientific conclusions are unaffected. The original publication has also been updated.
